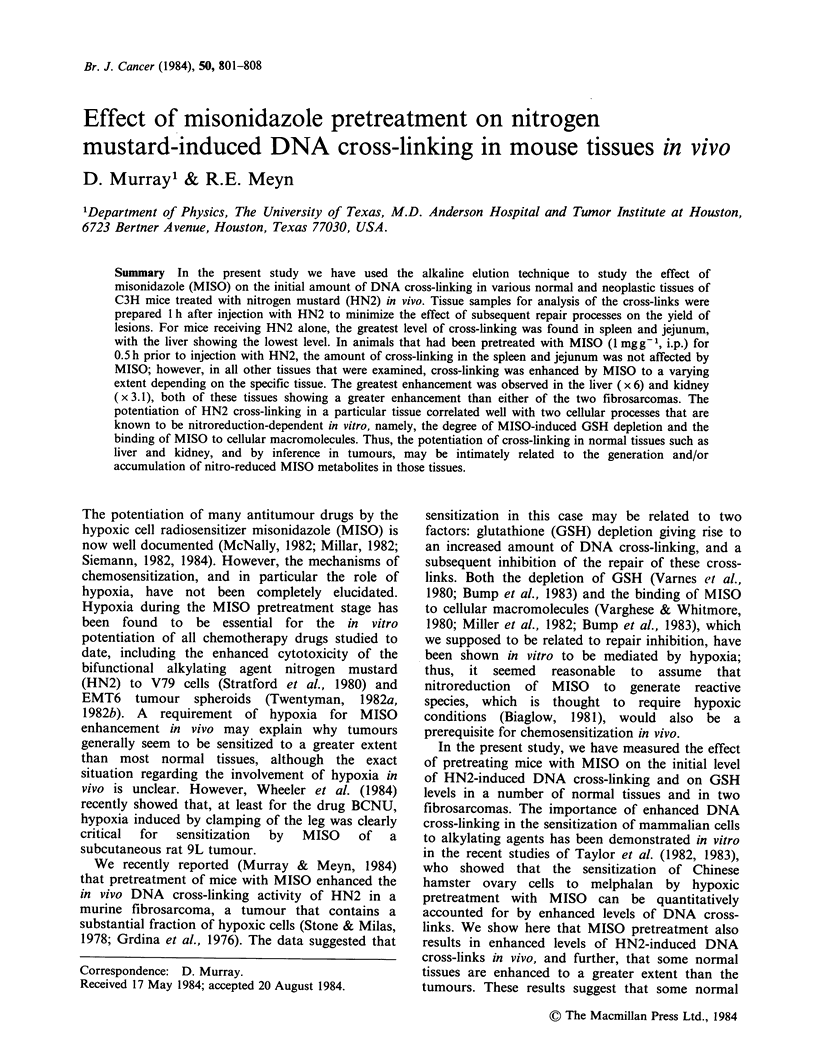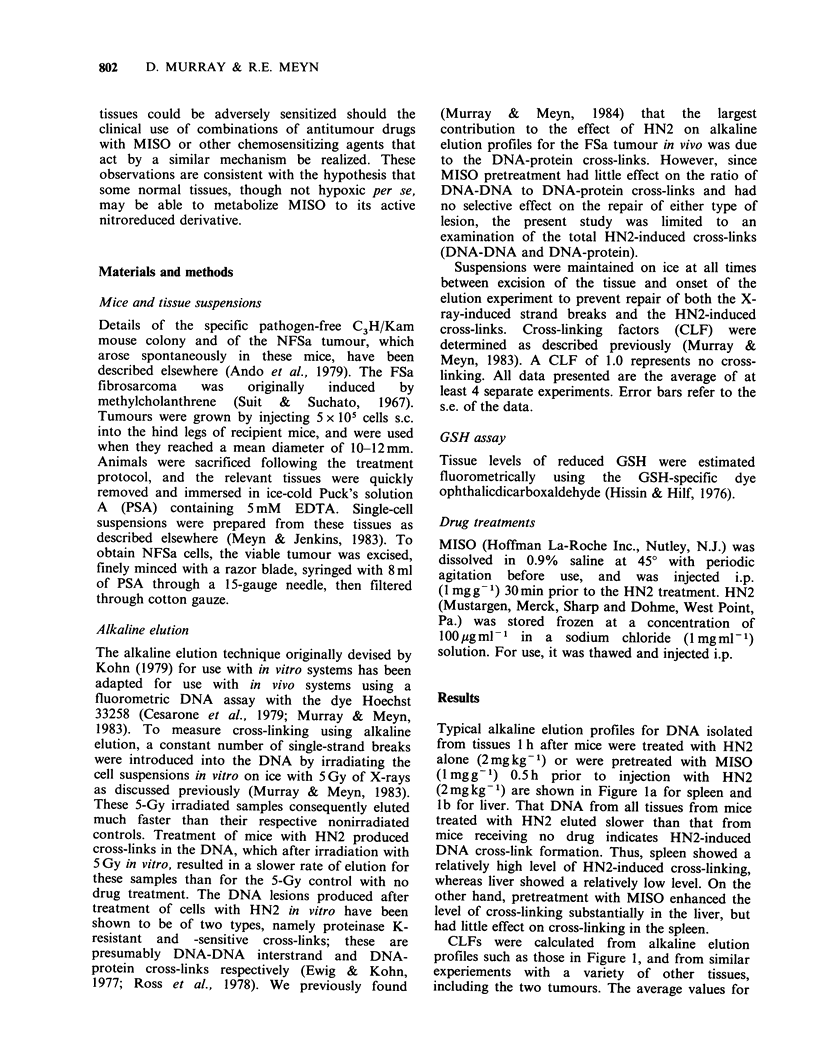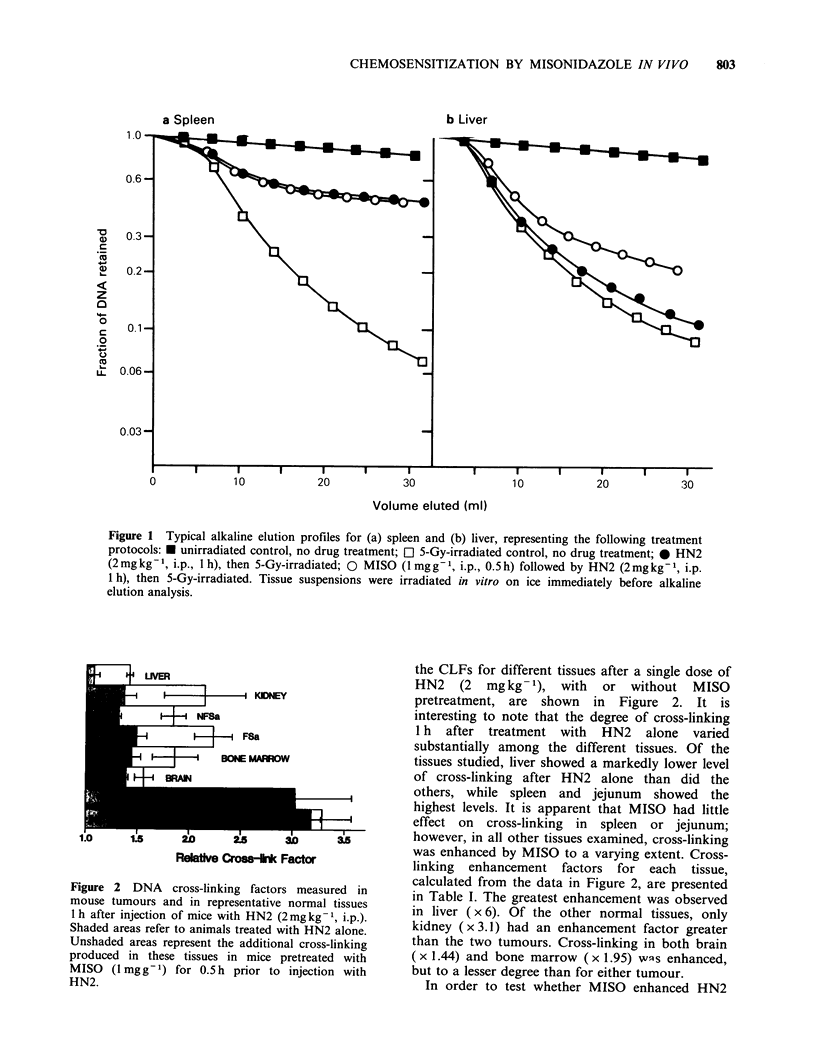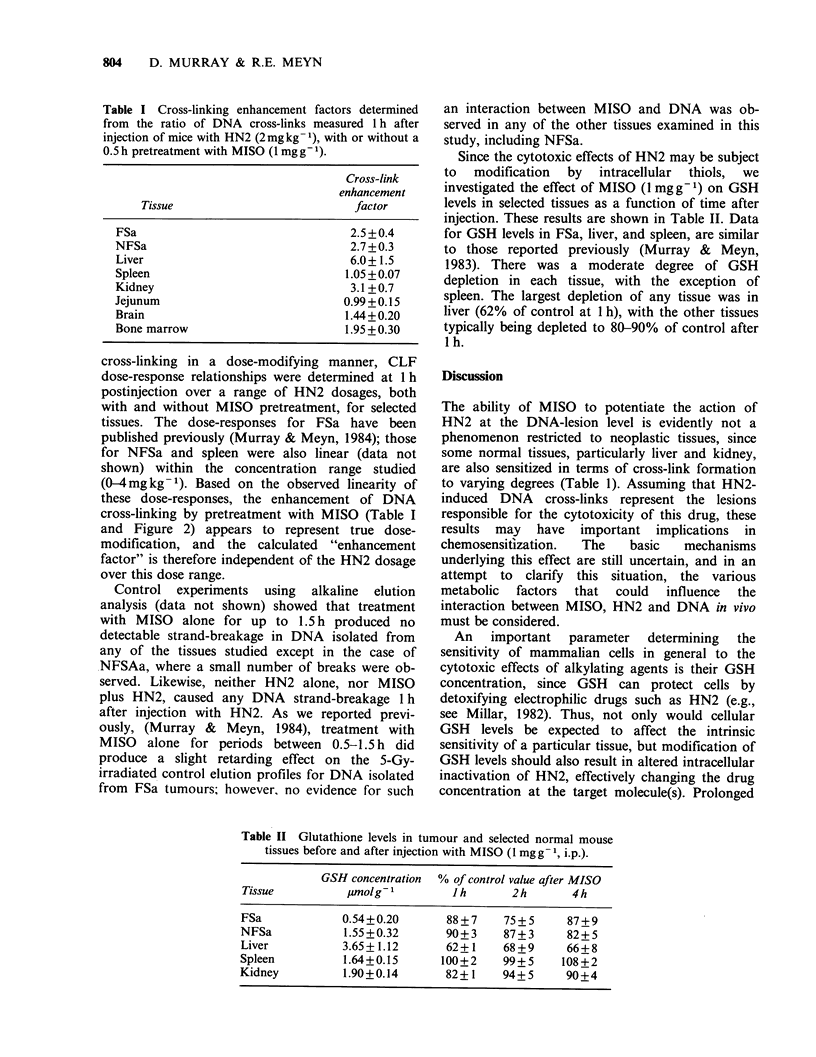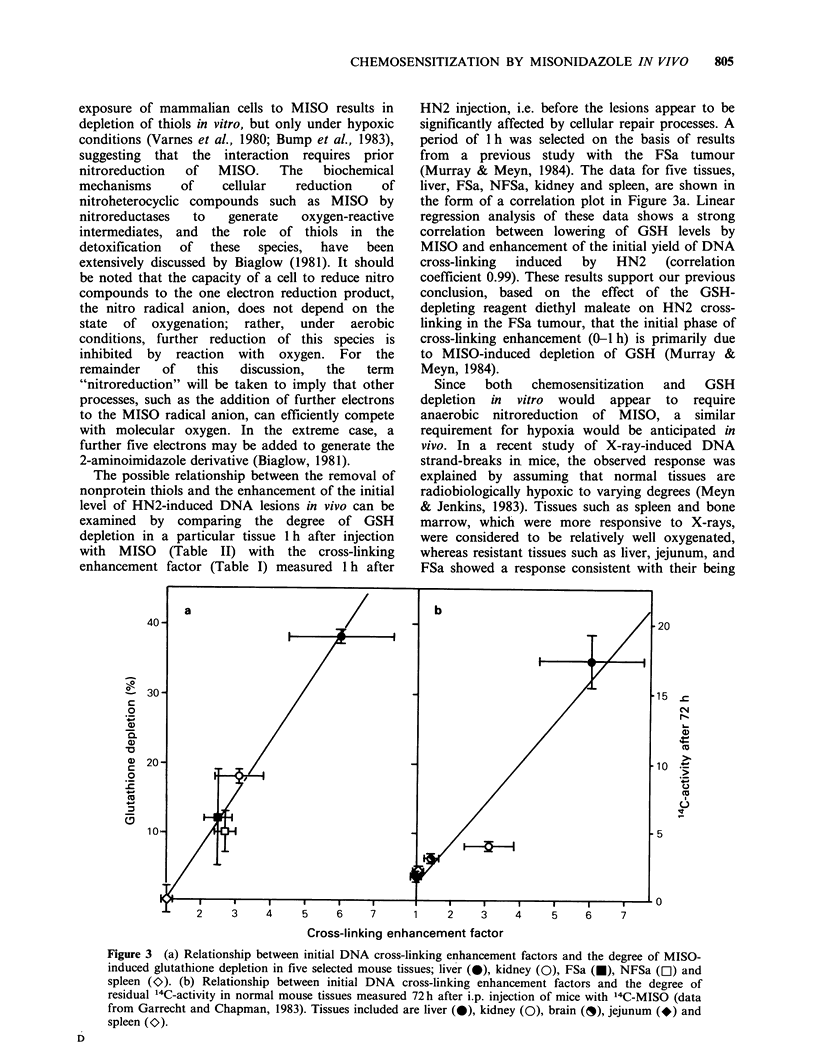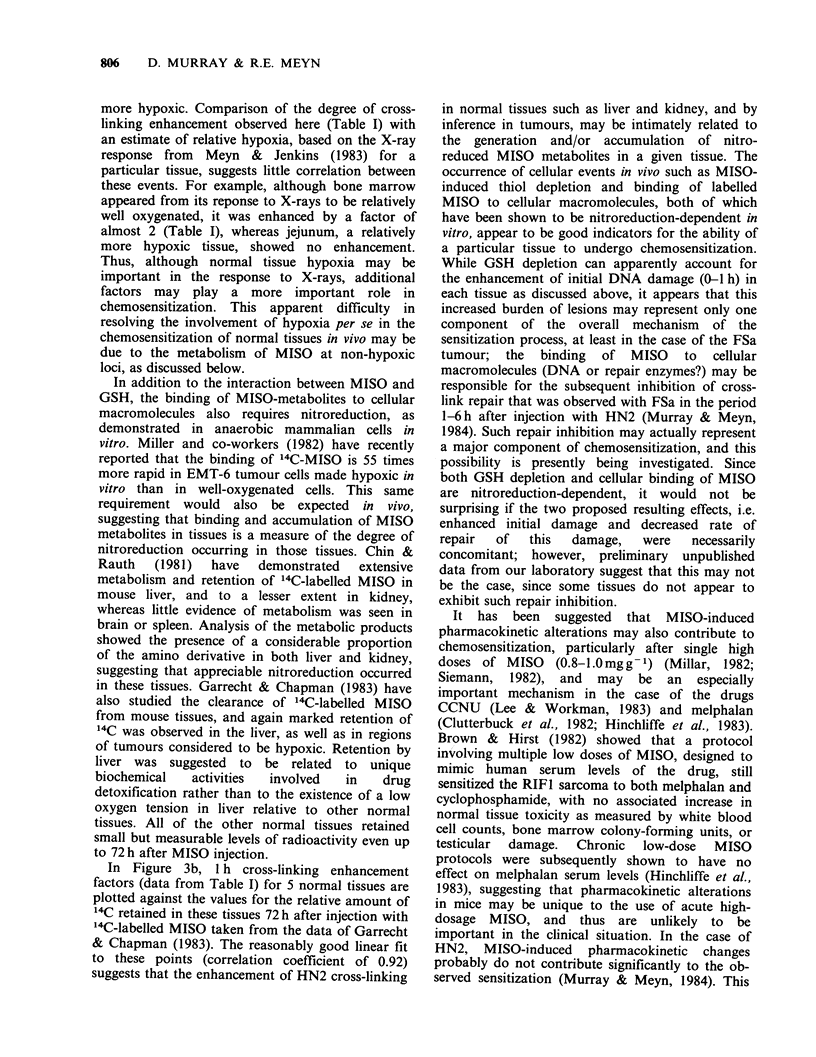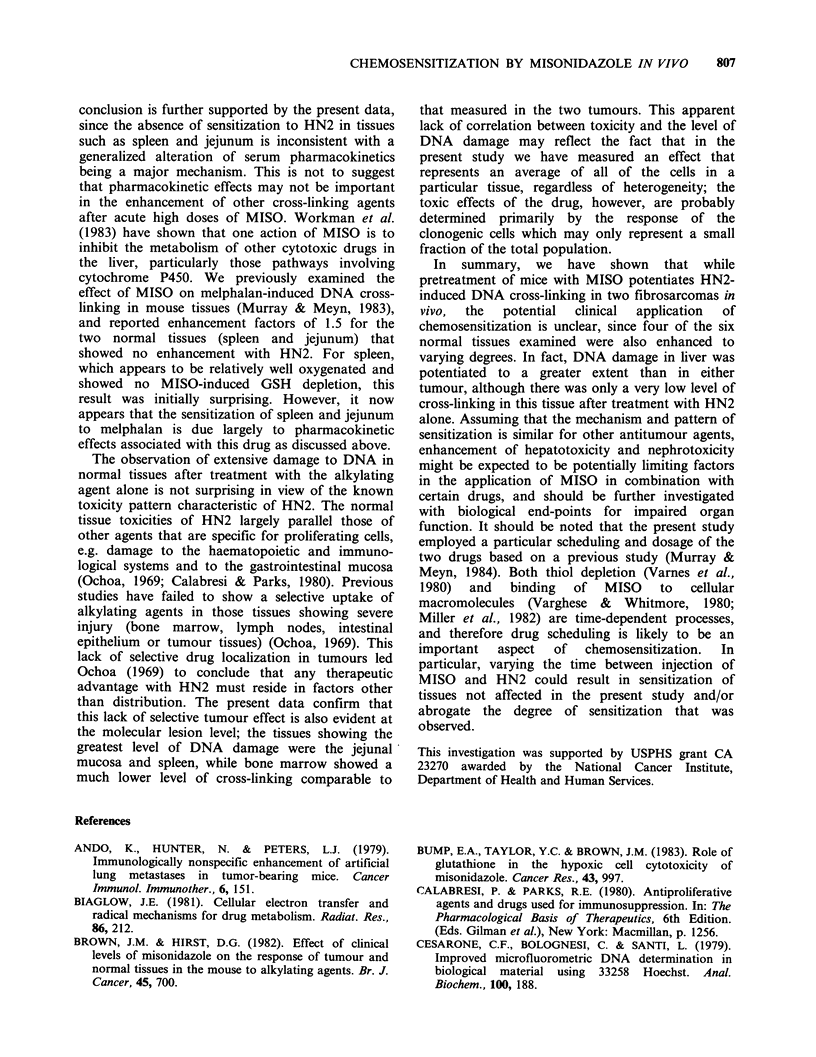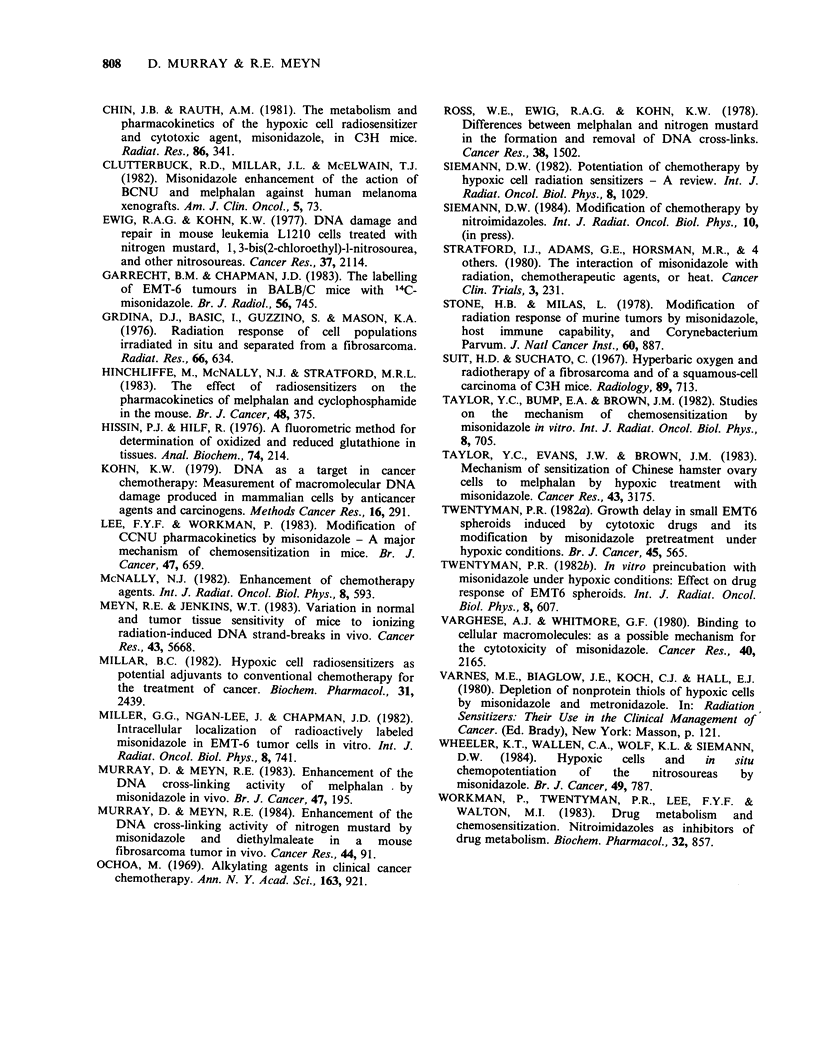# Effect of misonidazole pretreatment on nitrogen mustard-induced DNA cross-linking in mouse tissues in vivo.

**DOI:** 10.1038/bjc.1984.259

**Published:** 1984-12

**Authors:** D. Murray, R. E. Meyn

## Abstract

In the present study we have used the alkaline elution technique to study the effect of misonidazole (MISO) on the initial amount of DNA cross-linking in various normal and neoplastic tissues of C3H mice treated with nitrogen mustard (HN2) in vivo. Tissue samples for analysis of the cross-links were prepared 1 h after injection with HN2 to minimize the effect of subsequent repair processes on the yield of lesions. For mice receiving HN2 alone, the greatest level of cross-linking was found in spleen and jejunum, with the liver showing the lowest level. In animals that had been pretreated with MISO (1 mg g-1, i.p.) for 0.5 h prior to injection with HN2, the amount of cross-linking in the spleen and jejunum was not affected by MISO; however, in all other tissues that were examined, cross-linking was enhanced by MISO to a varying extent depending on the specific tissue. The greatest enhancement was observed in the liver (X 6) and kidney (X 3.1), both of these tissues showing a greater enhancement than either of the two fibrosarcomas. The potentiation of HN2 cross-linking in a particular tissue correlated well with two cellular processes that are known to be nitroreduction-dependent in vitro, namely, the degree of MISO-induced GSH depletion and the binding of MISO to cellular macromolecules. Thus, the potentiation of cross-linking in normal tissues such as liver and kidney, and by inference in tumours, may be intimately related to the generation and/or accumulation of nitro-reduced MISO metabolites in those tissues.


*Br. J. Cancer* (1984), **50**, 801–808

### Effect of misonidazole pretreatment on nitrogen mustard-induced DNA cross-linking in mouse tissues *in vivo*

D. Murray[1] & R.E. Meyn

[1]*Department of Physics, The University of Texas, M.D. Anderson Hospital and Tumor Institute at Houston, 6723 Bertner Avenue, Houston, Texas 77030, USA.*

**Summary** In the present study we have used the alkaline elution technique to study the effect of misonidazole (MISO) on the initial amount of DNA cross-linking in various normal and neoplastic tissues of C3H mice treated with nitrogen mustard (HN2) *in vivo*. Tissue samples for analysis of the cross-links were prepared 1 h after injection with HN2 to minimize the effect of subsequent repair processes on the yield of lesions. For mice receiving HN2 alone, the greatest level of cross-linking was found in spleen and jejunum, with the liver showing the lowest level. In animals that had been pretreated with MISO ($1\,mg\,g^{-1}$, i.p.) for 0.5 h prior to injection with HN2, the amount of cross-linking in the spleen and jejunum was not affected by MISO; however, in all other tissues that were examined, cross-linking was enhanced by MISO to a varying extent depending on the specific tissue. The greatest enhancement was observed in the liver ($\times 6$) and kidney ($\times 3.1$), both of these tissues showing a greater enhancement than either of the two fibrosarcomas. The potentiation of HN2 cross-linking in a particular tissue correlated well with two cellular processes that are known to be nitroreduction-dependent *in vitro*, namely, the degree of MISO-induced GSH depletion and the binding of MISO to cellular macromolecules. Thus, the potentiation of cross-linking in normal tissues such as liver and kidney, and by inference in tumours, may be intimately related to the generation and/or accumulation of nitro-reduced MISO metabolites in those tissues.

The potentiation of many antitumour drugs by the hypoxic cell radiosensitizer misonidazole (MISO) is now well documented (McNally, 1982; Millar, 1982; Siemann, 1982, 1984). However, the mechanisms of chemosensitization, and in particular the role of hypoxia, have not been completely elucidated. Hypoxia during the MISO pretreatment stage has been found to be essential for the *in vitro* potentiation of all chemotherapy drugs studied to date, including the enhanced cytotoxicity of the bifunctional alkylating agent nitrogen mustard (HN2) to V79 cells (Stratford *et al.*, 1980) and EMT6 tumour spheroids (Twentyman, 1982*a*, 1982*b*). A requirement of hypoxia for MISO enhancement *in vivo* may explain why tumours generally seem to be sensitized to a greater extent than most normal tissues, although the exact situation regarding the involvement of hypoxia *in vivo* is unclear. However, Wheeler *et al.* (1984) recently showed that, at least for the drug BCNU, hypoxia induced by clamping of the leg was clearly critical for sensitization by MISO of a subcutaneous rat 9L tumour.

We recently reported (Murray & Meyn, 1984) that pretreatment of mice with MISO enhanced the *in vivo* DNA cross-linking activity of HN2 in a murine fibrosarcoma, a tumour that contains a substantial fraction of hypoxic cells (Stone & Milas, 1978; Grdina *et al.*, 1976). The data suggested that sensitization in this case may be related to two factors: glutathione (GSH) depletion giving rise to an increased amount of DNA cross-linking, and a subsequent inhibition of the repair of these cross-links. Both the depletion of GSH (Varnes *et al.*, 1980; Bump *et al.*, 1983) and the binding of MISO to cellular macromolecules (Varghese & Whitmore, 1980; Miller *et al.*, 1982; Bump *et al.*, 1983), which we supposed to be related to repair inhibition, have been shown *in vitro* to be mediated by hypoxia; thus, it seemed reasonable to assume that nitroreduction of MISO to generate reactive species, which is thought to require hypoxic conditions (Biaglow, 1981), would also be a prerequisite for chemosensitization *in vivo*.

In the present study, we have measured the effect of pretreating mice with MISO on the initial level of HN2-induced DNA cross-linking and on GSH levels in a number of normal tissues and in two fibrosarcomas. The importance of enhanced DNA cross-linking in the sensitization of mammalian cells to alkylating agents has been demonstrated *in vitro* in the recent studies of Taylor *et al.* (1982, 1983), who showed that the sensitization of Chinese hamster ovary cells to melphalan by hypoxic pretreatment with MISO can be quantitatively accounted for by enhanced levels of DNA cross-links. We show here that MISO pretreatment also results in enhanced levels of HN2-induced DNA cross-links *in vivo*, and further, that some normal tissues are enhanced to a greater extent than the tumours. These results suggest that some normal

Correspondence: D. Murray.
Received 17 May 1984; accepted 20 August 1984.

© The Macmillan Press Ltd., 1984

tissues could be adversely sensitized should the clinical use of combinations of antitumour drugs with MISO or other chemosensitizing agents that act by a similar mechanism be realized. These observations are consistent with the hypothesis that some normal tissues, though not hypoxic *per se*, may be able to metabolize MISO to its active nitroreduced derivative.

### Materials and methods

### *Mice and tissue suspensions*

Details of the specific pathogen-free $C_3H$/Kam mouse colony and of the NFSa tumour, which arose spontaneously in these mice, have been described elsewhere (Ando *et al.*, 1979). The FSa fibrosarcoma was originally induced by methylcholanthrene (Suit & Suchato, 1967). Tumours were grown by injecting $5 \times 10^5$ cells s.c. into the hind legs of recipient mice, and were used when they reached a mean diameter of 10–12 mm. Animals were sacrificed following the treatment protocol, and the relevant tissues were quickly removed and immersed in ice-cold Puck's solution A (PSA) containing 5 mM EDTA. Single-cell suspensions were prepared from these tissues as described elsewhere (Meyn & Jenkins, 1983). To obtain NFSa cells, the viable tumour was excised, finely minced with a razor blade, syringed with 8 ml of PSA through a 15-gauge needle, then filtered through cotton gauze.

### *Alkaline elution*

The alkaline elution technique originally devised by Kohn (1979) for use with *in vitro* systems has been adapted for use with *in vivo* systems using a fluorometric DNA assay with the dye Hoechst 33258 (Cesarone *et al.*, 1979; Murray & Meyn, 1983). To measure cross-linking using alkaline elution, a constant number of single-strand breaks were introduced into the DNA by irradiating the cell suspensions *in vitro* on ice with 5 Gy of X-rays as discussed previously (Murray & Meyn, 1983). These 5-Gy irradiated samples consequently eluted much faster than their respective nonirradiated controls. Treatment of mice with HN2 produced cross-links in the DNA, which after irradiation with 5 Gy *in vitro*, resulted in a slower rate of elution for these samples than for the 5-Gy control with no drug treatment. The DNA lesions produced after treatment of cells with HN2 *in vitro* have been shown to be of two types, namely proteinase K-resistant and -sensitive cross-links; these are presumably DNA-DNA interstrand and DNA-protein cross-links respectively (Ewig & Kohn, 1977; Ross *et al.*, 1978). We previously found (Murray & Meyn, 1984) that the largest contribution to the effect of HN2 on alkaline elution profiles for the FSa tumour *in vivo* was due to the DNA-protein cross-links. However, since MISO pretreatment had little effect on the ratio of DNA-DNA to DNA-protein cross-links and had no selective effect on the repair of either type of lesion, the present study was limited to an examination of the total HN2-induced cross-links (DNA-DNA and DNA-protein).

Suspensions were maintained on ice at all times between excision of the tissue and onset of the elution experiment to prevent repair of both the X-ray-induced strand breaks and the HN2-induced cross-links. Cross-linking factors (CLF) were determined as described previously (Murray & Meyn, 1983). A CLF of 1.0 represents no cross-linking. All data presented are the average of at least 4 separate experiments. Error bars refer to the s.e. of the data.

### *GSH assay*

Tissue levels of reduced GSH were estimated fluorometrically using the GSH-specific dye ophthalicdicarboxaldehyde (Hissin & Hilf, 1976).

### *Drug treatments*

MISO (Hoffman La-Roche Inc., Nutley, N.J.) was dissolved in 0.9% saline at 45° with periodic agitation before use, and was injected i.p. ($1\,mg\,g^{-1}$) 30 min prior to the HN2 treatment. HN2 (Mustargen, Merck, Sharp and Dohme, West Point, Pa.) was stored frozen at a concentration of $100\,\mu g\,ml^{-1}$ in a sodium chloride ($1\,mg\,ml^{-1}$) solution. For use, it was thawed and injected i.p.

### Results

Typical alkaline elution profiles for DNA isolated from tissues 1 h after mice were treated with HN2 alone ($2\,mg\,kg^{-1}$) or were pretreated with MISO ($1\,mg\,g^{-1}$) 0.5 h prior to injection with HN2 ($2\,mg\,kg^{-1}$) are shown in Figure 1a for spleen and 1b for liver. That DNA from all tissues from mice treated with HN2 eluted slower than that from mice receiving no drug indicates HN2-induced DNA cross-link formation. Thus, spleen showed a relatively high level of HN2-induced cross-linking, whereas liver showed a relatively low level. On the other hand, pretreatment with MISO enhanced the level of cross-linking substantially in the liver, but had little effect on cross-linking in the spleen.

CLFs were calculated from alkaline elution profiles such as those in Figure 1, and from similar experiements with a variety of other tissues, including the two tumours. The average values for

**Figure 1** Typical alkaline elution profiles for (a) spleen and (b) liver, representing the following treatment protocols: ■ unirradiated control, no drug treatment; □ 5-Gy-irradiated control, no drug treatment; ● HN2 ($2\,mg\,kg^{-1}$, i.p., 1 h), then 5-Gy-irradiated; ○ MISO ($1\,mg\,g^{-1}$, i.p., 0.5 h) followed by HN2 ($2\,mg\,kg^{-1}$, i.p. 1 h), then 5-Gy-irradiated. Tissue suspensions were irradiated *in vitro* on ice immediately before alkaline elution analysis.

**Figure 2** DNA cross-linking factors measured in mouse tumours and in representative normal tissues 1 h after injection of mice with HN2 ($2\,mg\,kg^{-1}$, i.p.). Shaded areas refer to animals treated with HN2 alone. Unshaded areas represent the additional cross-linking produced in these tissues in mice pretreated with MISO ($1\,mg\,g^{-1}$) for 0.5 h prior to injection with HN2.

the CLFs for different tissues after a single dose of HN2 ($2\,mg\,kg^{-1}$), with or without MISO pretreatment, are shown in Figure 2. It is interesting to note that the degree of cross-linking 1 h after treatment with HN2 alone varied substantially among the different tissues. Of the tissues studied, liver showed a markedly lower level of cross-linking after HN2 alone than did the others, while spleen and jejunum showed the highest levels. It is apparent that MISO had little effect on cross-linking in spleen or jejunum; however, in all other tissues examined, cross-linking was enhanced by MISO to a varying extent. Cross-linking enhancement factors for each tissue, calculated from the data in Figure 2, are presented in Table I. The greatest enhancement was observed in liver (×6). Of the other normal tissues, only kidney (×3.1) had an enhancement factor greater than the two tumours. Cross-linking in both brain (×1.44) and bone marrow (×1.95) was enhanced, but to a lesser degree than for either tumour.

In order to test whether MISO enhanced HN2

**Table I** Cross-linking enhancement factors determined from the ratio of DNA cross-links measured 1 h after injection of mice with HN2 ($2\,mg\,kg^{-1}$), with or without a 0.5 h pretreatment with MISO ($1\,mg\,g^{-1}$).

| *Tissue* | *Cross-link enhancement factor* |
|---|---|
| FSa | 2.5±0.4 |
| NFSa | 2.7±0.3 |
| Liver | 6.0±1.5 |
| Spleen | 1.05±0.07 |
| Kidney | 3.1±0.7 |
| Jejunum | 0.99±0.15 |
| Brain | 1.44±0.20 |
| Bone marrow | 1.95±0.30 |

cross-linking in a dose-modifying manner, CLF dose-response relationships were determined at 1 h postinjection over a range of HN2 dosages, both with and without MISO pretreatment, for selected tissues. The dose-responses for FSa have been published previously (Murray & Meyn, 1984); those for NFSa and spleen were also linear (data not shown) within the concentration range studied (0–4 $mg\,kg^{-1}$). Based on the observed linearity of these dose-responses, the enhancement of DNA cross-linking by pretreatment with MISO (Table I and Figure 2) appears to represent true dose-modification, and the calculated "enhancement factor" is therefore independent of the HN2 dosage over this dose range.

Control experiments using alkaline elution analysis (data not shown) showed that treatment with MISO alone for up to 1.5 h produced no detectable strand-breakage in DNA isolated from any of the tissues studied except in the case of NFSAa, where a small number of breaks were observed. Likewise, neither HN2 alone, nor MISO plus HN2, caused any DNA strand-breakage 1 h after injection with HN2. As we reported previously, (Murray & Meyn, 1984), treatment with MISO alone for periods between 0.5–1.5 h did produce a slight retarding effect on the 5-Gy-irradiated control elution profiles for DNA isolated from FSa tumours; however, no evidence for such an interaction between MISO and DNA was observed in any of the other tissues examined in this study, including NFSa.

Since the cytotoxic effects of HN2 may be subject to modification by intracellular thiols, we investigated the effect of MISO ($1\,mg\,g^{-1}$) on GSH levels in selected tissues as a function of time after injection. These results are shown in Table II. Data for GSH levels in FSa, liver, and spleen, are similar to those reported previously (Murray & Meyn, 1983). There was a moderate degree of GSH depletion in each tissue, with the exception of spleen. The largest depletion of any tissue was in liver (62% of control at 1 h), with the other tissues typically being depleted to 80–90% of control after 1 h.

### Discussion

The ability of MISO to potentiate the action of HN2 at the DNA-lesion level is evidently not a phenomenon restricted to neoplastic tissues, since some normal tissues, particularly liver and kidney, are also sensitized in terms of cross-link formation to varying degrees (Table 1). Assuming that HN2-induced DNA cross-links represent the lesions responsible for the cytotoxicity of this drug, these results may have important implications in chemosensitization. The basic mechanisms underlying this effect are still uncertain, and in an attempt to clarify this situation, the various metabolic factors that could influence the interaction between MISO, HN2 and DNA *in vivo* must be considered.

An important parameter determining the sensitivity of mammalian cells in general to the cytotoxic effects of alkylating agents is their GSH concentration, since GSH can protect cells by detoxifying electrophilic drugs such as HN2 (e.g., see Millar, 1982). Thus, not only would cellular GSH levels be expected to affect the intrinsic sensitivity of a particular tissue, but modification of GSH levels should also result in altered intracellular inactivation of HN2, effectively changing the drug concentration at the target molecule(s). Prolonged

**Table II** Glutathione levels in tumour and selected normal mouse tissues before and after injection with MISO ($1\,mg\,g^{-1}$, i.p.).

| *Tissue* | *GSH concentration* $\mu mol\,g^{-1}$ | *% of control value after MISO* *1 h* | *2 h* | *4 h* |
|---|---|---|---|---|
| FSa | 0.54±0.20 | 88±7 | 75±5 | 87±9 |
| NFSa | 1.55±0.32 | 90±3 | 87±3 | 82±5 |
| Liver | 3.65±1.12 | 62±1 | 68±9 | 66±8 |
| Spleen | 1.64±0.15 | 100±2 | 99±5 | 108±2 |
| Kidney | 1.90±0.14 | 82±1 | 94±5 | 90±4 |

exposure of mammalian cells to MISO results in depletion of thiols *in vitro*, but only under hypoxic conditions (Varnes *et al.*, 1980; Bump *et al.*, 1983), suggesting that the interaction requires prior nitroreduction of MISO. The biochemical mechanisms of cellular reduction of nitroheterocyclic compounds such as MISO by nitroreductases to generate oxygen-reactive intermediates, and the role of thiols in the detoxification of these species, have been extensively discussed by Biaglow (1981). It should be noted that the capacity of a cell to reduce nitro compounds to the one electron reduction product, the nitro radical anion, does not depend on the state of oxygenation; rather, under aerobic conditions, further reduction of this species is inhibited by reaction with oxygen. For the remainder of this discussion, the term "nitroreduction" will be taken to imply that other processes, such as the addition of further electrons to the MISO radical anion, can efficiently compete with molecular oxygen. In the extreme case, a further five electrons may be added to generate the 2-aminoimidazole derivative (Biaglow, 1981).

The possible relationship between the removal of nonprotein thiols and the enhancement of the initial level of HN2-induced DNA lesions *in vivo* can be examined by comparing the degree of GSH depletion in a particular tissue 1 h after injection with MISO (Table II) with the cross-linking enhancement factor (Table I) measured 1 h after HN2 injection, i.e. before the lesions appear to be significantly affected by cellular repair processes. A period of 1 h was selected on the basis of results from a previous study with the FSa tumour (Murray & Meyn, 1984). The data for five tissues, liver, FSa, NFSa, kidney and spleen, are shown in the form of a correlation plot in Figure 3a. Linear regression analysis of these data shows a strong correlation between lowering of GSH levels by MISO and enhancement of the initial yield of DNA cross-linking induced by HN2 (correlation coefficient 0.99). These results support our previous conclusion, based on the effect of the GSH-depleting reagent diethyl maleate on HN2 cross-linking in the FSa tumour, that the initial phase of cross-linking enhancement (0–1 h) is primarily due to MISO-induced depletion of GSH (Murray & Meyn, 1984).

Since both chemosensitization and GSH depletion *in vitro* would appear to require anaerobic nitroreduction of MISO, a similar requirement for hypoxia would be anticipated *in vivo*. In a recent study of X-ray-induced DNA strand-breaks in mice, the observed response was explained by assuming that normal tissues are radiobiologically hypoxic to varying degrees (Meyn & Jenkins, 1983). Tissues such as spleen and bone marrow, which were more responsive to X-rays, were considered to be relatively well oxygenated, whereas resistant tissues such as liver, jejunum, and FSa showed a response consistent with their being

**Figure 3** (a) Relationship between initial DNA cross-linking enhancement factors and the degree of MISO-induced glutathione depletion in five selected mouse tissues; liver (●), kidney (○), FSa (■), NFSa (□) and spleen (◇). (b) Relationship between initial DNA cross-linking enhancement factors and the degree of residual $^{14}$C-activity in normal mouse tissues measured 72 h after i.p. injection of mice with $^{14}$C-MISO (data from Garrecht and Chapman, 1983). Tissues included are liver (●), kidney (○), brain (◐), jejunum (◆) and spleen (◇).

more hypoxic. Comparison of the degree of cross-linking enhancement observed here (Table I) with an estimate of relative hypoxia, based on the X-ray response from Meyn & Jenkins (1983) for a particular tissue, suggests little correlation between these events. For example, although bone marrow appeared from its reponse to X-rays to be relatively well oxygenated, it was enhanced by a factor of almost 2 (Table I), whereas jejunum, a relatively more hypoxic tissue, showed no enhancement. Thus, although normal tissue hypoxia may be important in the response to X-rays, additional factors may play a more important role in chemosensitization. This apparent difficulty in resolving the involvement of hypoxia *per se* in the chemosensitization of normal tissues *in vivo* may be due to the metabolism of MISO at non-hypoxic loci, as discussed below.

In addition to the interaction between MISO and GSH, the binding of MISO-metabolites to cellular macromolecules also requires nitroreduction, as demonstrated in anaerobic mammalian cells *in vitro*. Miller and co-workers (1982) have recently reported that the binding of $^{14}$C-MISO is 55 times more rapid in EMT-6 tumour cells made hypoxic *in vitro* than in well-oxygenated cells. This same requirement would also be expected *in vivo*, suggesting that binding and accumulation of MISO metabolites in tissues is a measure of the degree of nitroreduction occurring in those tissues. Chin & Rauth (1981) have demonstrated extensive metabolism and retention of $^{14}$C-labelled MISO in mouse liver, and to a lesser extent in kidney, whereas little evidence of metabolism was seen in brain or spleen. Analysis of the metabolic products showed the presence of a considerable proportion of the amino derivative in both liver and kidney, suggesting that appreciable nitroreduction occurred in these tissues. Garrecht & Chapman (1983) have also studied the clearance of $^{14}$C-labelled MISO from mouse tissues, and again marked retention of $^{14}$C was observed in the liver, as well as in regions of tumours considered to be hypoxic. Retention by liver was suggested to be related to unique biochemical activities involved in drug detoxification rather than to the existence of a low oxygen tension in liver relative to other normal tissues. All of the other normal tissues retained small but measurable levels of radioactivity even up to 72 h after MISO injection.

In Figure 3b, 1 h cross-linking enhancement factors (data from Table I) for 5 normal tissues are plotted against the values for the relative amount of $^{14}$C retained in these tissues 72 h after injection with $^{14}$C-labelled MISO taken from the data of Garrecht & Chapman (1983). The reasonably good linear fit to these points (correlation coefficient of 0.92) suggests that the enhancement of HN2 cross-linking in normal tissues such as liver and kidney, and by inference in tumours, may be intimately related to the generation and/or accumulation of nitro-reduced MISO metabolites in a given tissue. The occurrence of cellular events *in vivo* such as MISO-induced thiol depletion and binding of labelled MISO to cellular macromolecules, both of which have been shown to be nitroreduction-dependent *in vitro*, appear to be good indicators for the ability of a particular tissue to undergo chemosensitization. While GSH depletion can apparently account for the enhancement of initial DNA damage (0–1 h) in each tissue as discussed above, it appears that this increased burden of lesions may represent only one component of the overall mechanism of the sensitization process, at least in the case of the FSa tumour; the binding of MISO to cellular macromolecules (DNA or repair enzymes?) may be responsible for the subsequent inhibition of cross-link repair that was observed with FSa in the period 1–6 h after injection with HN2 (Murray & Meyn, 1984). Such repair inhibition may actually represent a major component of chemosensitization, and this possibility is presently being investigated. Since both GSH depletion and cellular binding of MISO are nitroreduction-dependent, it would not be surprising if the two proposed resulting effects, i.e. enhanced initial damage and decreased rate of repair of this damage, were necessarily concomitant; however, preliminary unpublished data from our laboratory suggest that this may not be the case, since some tissues do not appear to exhibit such repair inhibition.

It has been suggested that MISO-induced pharmacokinetic alterations may also contribute to chemosensitization, particularly after single high doses of MISO (0.8–1.0 mg g$^{-1}$) (Millar, 1982; Siemann, 1982), and may be an especially important mechanism in the case of the drugs CCNU (Lee & Workman, 1983) and melphalan (Clutterbuck *et al.*, 1982; Hinchliffe *et al.*, 1983). Brown & Hirst (1982) showed that a protocol involving multiple low doses of MISO, designed to mimic human serum levels of the drug, still sensitized the RIF1 sarcoma to both melphalan and cyclophosphamide, with no associated increase in normal tissue toxicity as measured by white blood cell counts, bone marrow colony-forming units, or testicular damage. Chronic low-dose MISO protocols were subsequently shown to have no effect on melphalan serum levels (Hinchliffe *et al.*, 1983), suggesting that pharmacokinetic alterations in mice may be unique to the use of acute high-dosage MISO, and thus are unlikely to be important in the clinical situation. In the case of HN2, MISO-induced pharmacokinetic changes probably do not contribute significantly to the observed sensitization (Murray & Meyn, 1984). This conclusion is further supported by the present data, since the absence of sensitization to HN2 in tissues such as spleen and jejunum is inconsistent with a generalized alteration of serum pharmacokinetics being a major mechanism. This is not to suggest that pharmacokinetic effects may not be important in the enhancement of other cross-linking agents after acute high doses of MISO. Workman *et al.* (1983) have shown that one action of MISO is to inhibit the metabolism of other cytotoxic drugs in the liver, particularly those pathways involving cytochrome P450. We previously examined the effect of MISO on melphalan-induced DNA cross-linking in mouse tissues (Murray & Meyn, 1983), and reported enhancement factors of 1.5 for the two normal tissues (spleen and jejunum) that showed no enhancement with HN2. For spleen, which appears to be relatively well oxygenated and showed no MISO-induced GSH depletion, this result was initially surprising. However, it now appears that the sensitization of spleen and jejunum to melphalan is due largely to pharmacokinetic effects associated with this drug as discussed above.

The observation of extensive damage to DNA in normal tissues after treatment with the alkylating agent alone is not surprising in view of the known toxicity pattern characteristic of HN2. The normal tissue toxicities of HN2 largely parallel those of other agents that are specific for proliferating cells, e.g. damage to the haematopoietic and immunological systems and to the gastrointestinal mucosa (Ochoa, 1969; Calabresi & Parks, 1980). Previous studies have failed to show a selective uptake of alkylating agents in those tissues showing severe injury (bone marrow, lymph nodes, intestinal epithelium or tumour tissues) (Ochoa, 1969). This lack of selective drug localization in tumours led Ochoa (1969) to conclude that any therapeutic advantage with HN2 must reside in factors other than distribution. The present data confirm that this lack of selective tumour effect is also evident at the molecular lesion level; the tissues showing the greatest level of DNA damage were the jejunal mucosa and spleen, while bone marrow showed a much lower level of cross-linking comparable to that measured in the two tumours. This apparent lack of correlation between toxicity and the level of DNA damage may reflect the fact that in the present study we have measured an effect that represents an average of all of the cells in a particular tissue, regardless of heterogeneity; the toxic effects of the drug, however, are probably determined primarily by the response of the clonogenic cells which may only represent a small fraction of the total population.

In summary, we have shown that while pretreatment of mice with MISO potentiates HN2-induced DNA cross-linking in two fibrosarcomas *in vivo*, the potential clinical application of chemosensitization is unclear, since four of the six normal tissues examined were also enhanced to varying degrees. In fact, DNA damage in liver was potentiated to a greater extent than in either tumour, although there was only a very low level of cross-linking in this tissue after treatment with HN2 alone. Assuming that the mechanism and pattern of sensitization is similar for other antitumour agents, enhancement of hepatotoxicity and nephrotoxicity might be expected to be potentially limiting factors in the application of MISO in combination with certain drugs, and should be further investigated with biological end-points for impaired organ function. It should be noted that the present study employed a particular scheduling and dosage of the two drugs based on a previous study (Murray & Meyn, 1984). Both thiol depletion (Varnes *et al.*, 1980) and binding of MISO to cellular macromolecules (Varghese & Whitmore, 1980; Miller *et al.*, 1982) are time-dependent processes, and therefore drug scheduling is likely to be an important aspect of chemosensitization. In particular, varying the time between injection of MISO and HN2 could result in sensitization of tissues not affected in the present study and/or abrogate the degree of sensitization that was observed.

This investigation was supported by USPHS grant CA 23270 awarded by the National Cancer Institute, Department of Health and Human Services.

### References

ANDO, K., HUNTER, N. & PETERS, L.J. (1979). Immunologically nonspecific enhancement of artificial lung metastases in tumor-bearing mice. *Cancer Immunol. Immunother.*, **6**, 151.

BIAGLOW, J.E. (1981). Cellular electron transfer and radical mechanisms for drug metabolism. *Radiat. Res.*, **86**, 212.

BROWN, J.M. & HIRST, D.G. (1982). Effect of clinical levels of misonidazole on the response of tumour and normal tissues in the mouse to alkylating agents. *Br. J. Cancer*, **45**, 700.

BUMP, E.A., TAYLOR, Y.C. & BROWN, J.M. (1983). Role of glutathione in the hypoxic cell cytotoxicity of misonidazole. *Cancer Res.*, **43**, 997.

CALABRESI, P. & PARKS, R.E. (1980). Antiproliferative agents and drugs used for immunosuppression. In: *The Pharmacological Basis of Therapeutics*, 6th Edition. (Eds. Gilman *et al.*), New York: Macmillan, p. 1256.

CESARONE, C.F., BOLOGNESI, C. & SANTI, L. (1979). Improved microfluorometric DNA determination in biological material using 33258 Hoechst. *Anal. Biochem.*, **100**, 188.

CHIN, J.B. & RAUTH, A.M. (1981). The metabolism and pharmacokinetics of the hypoxic cell radiosensitizer and cytotoxic agent, misonidazole, in C3H mice. *Radiat. Res.*, **86**, 341.

CLUTTERBUCK, R.D., MILLAR, J.L. & McELWAIN, T.J. (1982). Misonidazole enhancement of the action of BCNU and melphalan against human melanoma xenografts. *Am. J. Clin. Oncol.*, **5**, 73.

EWIG, R.A.G. & KOHN, K.W. (1977). DNA damage and repair in mouse leukemia L1210 cells treated with nitrogen mustard, 1,3-bis(2-chloroethyl)-l-nitrosourea, and other nitrosoureas. *Cancer Res.*, **37**, 2114.

GARRECHT, B.M. & CHAPMAN, J.D. (1983). The labelling of EMT-6 tumours in BALB/C mice with $^{14}$C-misonidazole. *Br. J. Radiol.*, **56**, 745.

GRDINA, D.J., BASIC, I., GUZZINO, S. & MASON, K.A. (1976). Radiation response of cell populations irradiated in situ and separated from a fibrosarcoma. *Radiat. Res.*, **66**, 634.

HINCHLIFFE, M., McNALLY, N.J. & STRATFORD, M.R.L. (1983). The effect of radiosensitizers on the pharmacokinetics of melphalan and cyclophosphamide in the mouse. *Br. J. Cancer*, **48**, 375.

HISSIN, P.J. & HILF, R. (1976). A fluorometric method for determination of oxidized and reduced glutathione in tissues. *Anal. Biochem.*, **74**, 214.

KOHN, K.W. (1979). DNA as a target in cancer chemotherapy: Measurement of macromolecular DNA damage produced in mammalian cells by anticancer agents and carcinogens. *Methods Cancer Res.*, **16**, 291.

LEE, F.Y.F. & WORKMAN, P. (1983). Modification of CCNU pharmacokinetics by misonidazole – A major mechanism of chemosensitization in mice. *Br. J. Cancer*, **47**, 659.

McNALLY, N.J. (1982). Enhancement of chemotherapy agents. *Int. J. Radiat. Oncol. Biol. Phys.*, **8**, 593.

MEYN, R.E. & JENKINS, W.T. (1983). Variation in normal and tumor tissue sensitivity of mice to ionizing radiation-induced DNA strand-breaks in vivo. *Cancer Res.*, **43**, 5668.

MILLAR, B.C. (1982). Hypoxic cell radiosensitizers as potential adjuvants to conventional chemotherapy for the treatment of cancer. *Biochem. Pharmacol.*, **31**, 2439.

MILLER, G.G., NGAN-LEE, J. & CHAPMAN, J.D. (1982). Intracellular localization of radioactively labeled misonidazole in EMT-6 tumor cells in vitro. *Int. J. Radiat. Oncol. Biol. Phys.*, **8**, 741.

MURRAY, D. & MEYN, R.E. (1983). Enhancement of the DNA cross-linking activity of melphalan by misonidazole in vivo. *Br. J. Cancer*, **47**, 195.

MURRAY, D. & MEYN, R.E. (1984). Enhancement of the DNA cross-linking activity of nitrogen mustard by misonidazole and diethylmaleate in a mouse fibrosarcoma tumor in vivo. *Cancer Res.*, **44**, 91.

OCHOA, M. (1969). Alkylating agents in clinical cancer chemotherapy. *Ann. N. Y. Acad. Sci.*, **163**, 921.

ROSS, W.E., EWIG, R.A.G. & KOHN, K.W. (1978). Differences between melphalan and nitrogen mustard in the formation and removal of DNA cross-links. *Cancer Res.*, **38**, 1502.

SIEMANN, D.W. (1982). Potentiation of chemotherapy by hypoxic cell radiation sensitizers – A review. *Int. J. Radiat. Oncol. Biol. Phys.*, **8**, 1029.

SIEMANN, D.W. (1984). Modification of chemotherapy by nitroimidazoles. *Int. J. Radiat. Oncol. Biol. Phys.*, **10**, (in press).

STRATFORD, I.J., ADAMS, G.E., HORSMAN, M.R., & 4 others. (1980). The interaction of misonidazole with radiation, chemotherapeutic agents, or heat. *Cancer Clin. Trials*, **3**, 231.

STONE, H.B. & MILAS, L. (1978). Modification of radiation response of murine tumors by misonidazole, host immune capability, and Corynebacterium Parvum. *J. Natl Cancer Inst.*, **60**, 887.

SUIT, H.D. & SUCHATO, C. (1967). Hyperbaric oxygen and radiotherapy of a fibrosarcoma and of a squamous-cell carcinoma of C3H mice. *Radiology*, **89**, 713.

TAYLOR, Y.C., BUMP, E.A. & BROWN, J.M. (1982). Studies on the mechanism of chemosensitization by misonidazole *in vitro*. *Int. J. Radiat. Oncol. Biol. Phys.*, **8**, 705.

TAYLOR, Y.C., EVANS, J.W. & BROWN, J.M. (1983). Mechanism of sensitization of Chinese hamster ovary cells to melphalan by hypoxic treatment with misonidazole. *Cancer Res.*, **43**, 3175.

TWENTYMAN, P.R. (1982*a*). Growth delay in small EMT6 spheroids induced by cytotoxic drugs and its modification by misonidazole pretreatment under hypoxic conditions. *Br. J. Cancer*, **45**, 565.

TWENTYMAN, P.R. (1982*b*). *In vitro* preincubation with misonidazole under hypoxic conditions: Effect on drug response of EMT6 spheroids. *Int. J. Radiat. Oncol. Biol. Phys.*, **8**, 607.

VARGHESE, A.J. & WHITMORE, G.F. (1980). Binding to cellular macromolecules: as a possible mechanism for the cytotoxicity of misonidazole. *Cancer Res.*, **40**, 2165.

VARNES, M.E., BIAGLOW, J.E., KOCH, C.J. & HALL, E.J. (1980). Depletion of nonprotein thiols of hypoxic cells by misonidazole and metronidazole. In: *Radiation Sensitizers: Their Use in the Clinical Management of Cancer*. (Ed. Brady), New York: Masson, p. 121.

WHEELER, K.T., WALLEN, C.A., WOLF, K.L. & SIEMANN, D.W. (1984). Hypoxic cells and *in situ* chemopotentiation of the nitrosoureas by misonidazole. *Br. J. Cancer*, **49**, 787.

WORKMAN, P., TWENTYMAN, P.R., LEE, F.Y.F. & WALTON, M.I. (1983). Drug metabolism and chemosensitization. Nitroimidazoles as inhibitors of drug metabolism. *Biochem. Pharmacol.*, **32**, 857.